# A Comparative Study on Micellar and Solubilizing Behavior of Three EO-PO Based Star Block Copolymers Varying in Hydrophobicity and Their Application for the In Vitro Release of Anticancer Drugs

**DOI:** 10.3390/polym10010076

**Published:** 2018-01-15

**Authors:** Bijal Vyas, Sadafara A. Pillai, Anita Bahadur, Pratap Bahadur

**Affiliations:** 1 Department of Chemistry, Veer Narmad South Gujarat University, Surat 395007, India; vyasbijal80@gmail.com (B.V.); sadafarashirgar@gmail.com (S.A.P.); 2 Department of Zoology, PT Sarvajanik College of Science, Surat 395001, India; anita26p@gmail.com

**Keywords:** Tetronics, micelles, in vitro release, anticancer drugs, solubilization, drug delivery

## Abstract

The temperature and pH dependent self-assembly of three star shaped ethylene oxide-propylene oxide (EO-PO) block copolymers (Tetronics^®^ 304, 904 and 908) with widely different hydrophobicity was examined in aqueous solutions. Physico-chemical methods viz. viscosity, cloud point, solubilization along with thermal, scattering and spectral techniques shows strongly temperature and salt dependent solution behavior. T304 possessing low molecular weight did not form micelles; moderately hydrophilic T904 remained as micelles at ambient temperature and showed micellar growth while very hydrophilic T908 formed micelles at elevated temperatures. The surface activity/micellization/solubilization power was favored in the presence of salt. The copolymers turn more hydrophilic in acidic pH due to protonation of central ethylene diamine moiety that hinders micelle formation. The solubilization of a model insoluble azo dye 1-(o-Tolylazo)-2-naphthol (Orange OT) and hydrophobic drugs (quercetin and curcumin) for copolymer solutions in aqueous and salt solutions are also reported. Among the three copolymers, T904 showed maximum solubility of dye and drugs, hence the in vitro release of drugs from T904 micelles was estimated and the effect on cytotoxicity of loading the drugs in T904 micelles was compared with the cytotoxicity of free drugs on the CHO-K1 cells. The results from the present work provide a better insight in selection of Tetronics^®^ for their application in different therapeutic applications.

## 1. Introduction

Block copolymers contain at least two incompatible blocks and thus show micro domain formation in solid state and self-assembly in selective solvents; these properties coupled with advances in polymerization techniques have made polymeric surfactants highly useful materials [1,2,3]. Pluronics^®^ and Tetronics^®^ are commercially available poly(ethylene oxide) (PEO)-poly(propylene oxide) (PPO) block copolymers with unique temperature dependent micellization/surface activity and reversible thermorheological behavior that make these substances widely useful in cosmetic/detergents/food/pharmaceutical industries. Due to their nontoxicity and low immune response, some of these copolymers are now Food and Drug Administration (FDA) approved. Their emerging applications in fabrication of mesoporous materials [4,5], synthesis of nanoparticles [6,7] and as nanocarriers for drug delivery systems [8,9,10,11,12] have generated more interest in researchers [13,14]. Strongly temperature dependent micellization and gelation of these amphiphilic copolymers have been thoroughly examined in the last few decades though mostly on linear PEO–PPO–PEO triblock copolymers and there exist several reviews [1,15,16]. However, only a few authors have reported on the aqueous solution behavior of their star shaped counterparts [17,18,19,20,21,22,23,24,25,26,27,28,29,30].

**Figure 1:**
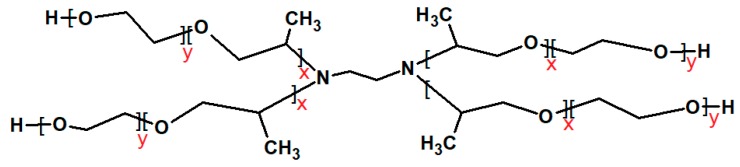
General structure of Tetronics^®^.

Tetronics^®^ (also known as poloxamines) present an X-shaped structure made of an ethylenediamine central group bonded to four chains of PPO–PEO blocks. Tetronics^®^ are synthesized by the sequential reaction of the acceptor ethylenediamine molecule first with propylene oxide (PO) and then with ethylene oxide (EO) precursors, resulting in a four-arm PEO-terminated molecular structure (Figure 1). The unique structure of Tetronics^®^ provides them with multistimulus responsiveness. In this context, the two tertiary amine central groups play an essential role in conferring thermodynamic stability and pH sensitivity. The micelle formation of Tetronics^®^ is slightly different from the Pluronics^®^ as the former also show some pH responsiveness. Like Pluronics^®^, a slight increase in the temperature can induce surface activity/micellization/gelation due to the dehydration of PPO and PEO blocks. The central diamine unit in Tetronics^®^ molecule is pH sensitive and can be protonated in acidic solution. Low pH and low temperature may thus hamper micellization. Albeit still limited, the studies on Tetronics^®^ have revealed their potential in different fields. These cover broad area of applications including petroleum industries where these are used in comparatively higher concentrations either as de-emulsifiers or as antifoaming agents [31,32], as an important ingredient in contact lens washing solutions [33,34], in pharmaceutical and biomedical field as constituents of transdermal formulations [35], in nanoparticle engineering [36] and as tissue scaffoldings [37,38]. De Lisi et al. [39] studied the self-assembly and oil solubilizing behavior of T1107 as a function of temperature and pH and revealed improved solubilization and oil induced micellization, which can be tuned further by varying temperature and pH. Larrañeta et al. [40] synthesized different types of gels by the addition of α-cyclodextrin in the T904 solutions for sustained drug delivery applications. González-Gaitano et al. [41] explored the effect of different native and modified cyclodextrins on T904 micelles and revealed that most substituted cyclodextrins induced micellar breakdown while native cyclodextrins promoted the formation of inclusion complexes. Recently, Poellmann [42] based on his study on T1107 with denatured hen egg white lysozyme found a potential application of T1107 as a synthetic chaperone that enhances the molecular repair phenomenon in cells. Gonzalez-Lopez et al. [20] explored the micellization of different Tetronics^®^ with varying structural features in acidic media. There are also contributions from our group concerning the effect of different additives on micellar behavior of Tetronics^®^ [43,44,45,46,47,48,49].

It can be understood from the above literature survey that reports on individual behavior of different Tetronics^®^ in the presence of variety of additives have been published in literature. However, a systematic study comparing the self-assembly of different Tetronics^®^ on the basis of their molecular architecture in aqueous and salt solutions is still missing, to the best of our knowledge. The knowledge attained from this work can be useful in anticipating the performance of the copolymer for the desired application. With this view point, we have tried to elucidate the aggregation behavior of three Tetronics^®^, mainly T304, T904 and T908 incorporated with different %EO (in case of T904 and T908, %PEO = 40 and 80) and with different molecular weight (in the case of T304 and T904, but with same %PEO = 40). This study was further extended by determining the solubility of a hydrophobic dye (orange-OT) and drugs (quercetin and curcumin) in micelles under different solution conditions. T904 with a moderately hydrophobic character showed a maximum solubility. Hence, in vitro release of the hydrophobic drugs from its micelles was measured and the influence on cytotoxicity of the drugs being loaded in micelles was compared with the free drugs. The findings of this work will be highly useful for the proper exploitation of copolymer micelles in several industrial and pharmaceutical applications.

## 2. Materials and Methods

Tetronics^®^ 304, 904 and 908 were gift samples from the BASF Corporation (Parsippany, NJ, USA) and were used as received. The structural formula for Tetronics^®^ block copolymers (Figure 1) and molecular/physico-chemical properties are shown in Table 1.

Sodium chloride (Merck, Mumbai, India, analytical grade), the drugs, quercetin and curcumin (Sigma Aldrich, Mumbai, India) and the dye, Orange-OT (TCI Chemicals, Chennai, Tamilnadu, India) were used as received. Solutions for dynamic light scattering (DLS) measurements were prepared in nano-pure water obtained from Millipore Milli-Q purification system (Mumbai, India). D_2_O (99.9%) obtained from Sigma Chemical Company (Mumbai, India) was used for nuclear magnetic resonance (NMR) and small-angle neutron scattering (SANS) measurements. The chinese hamster ovary (CHO-K1) cell line for the toxicity assay was procured from American Type Culture Collection (ATCC).

### 2.1. Methods

#### 2.1.1. Cloud Point (CP)

Cloud points were determined by visual observation of the turbidity of the solution (in 20 mL glass vials) immersed in a temperature controlled water bath. The solutions were stirred with a magnetic bar while being heated. All of the measured CP values were reproducible up to ±1.0 °C.

#### 2.1.2. Surface Tension

The surface tension measurements were done with a KRUSS Easy Tensiometer from Kruss Gmbh (Hamburg, Germany) using the Wilhelmy plate method. The surface tension of double distilled water 71.8 mN·m^−1^ at 25.0 ± 0.1 °C was used to calibrate the instrument. The surface tension of each solution was measured by successive additions of the stock solutions in double distilled water after thorough mixing and equilibration. The series of measurements were repeated at least three times. The reproducibility of surface tension measurements is estimated to be within ±0.2 mN·m^−1^.

#### 2.1.3. Viscosity

The viscosities of solutions were measured using an Ubbelohde suspended level capillary viscometer. The viscometer was suspended vertically in a thermostat at ±0.1 °C. A clean and dry viscometer was used for each measurement. The flow time (usually exceeding 170 s) of a constant volume of the solution through the capillary was used to calculate the viscosity of the solution.

#### 2.1.4. Nuclear Magnetic Resonance (NMR)

The ^1^H-NMR spectra were recorded on a Bruker DMX Avance 600 spectrometer (Osaka, Japan) over a wide temperature range. The sample temperature was kept constant within ±0.1 °C by using a Bruker BCU-05 temperature control unit. The samples were equilibrated at the desired temperature for at least 15 min prior to measurement.

#### 2.1.5. High-Sensitivity Differential Scanning Calorimetry (HSDSC)

Calorimetric measurements were carried out using a Microcal MC-2 instrument (Microcal Inc., Amherst, MA, USA) and the DA-2 dedicated software package (provided by Microcal, Malvern, UK) for data acquisition. Samples were equilibrated in the HSDSC cells for a minimum of 60 min prior to each run, and scans performed at a scan rate of 60 Kh^−1^.

#### 2.1.6. Dynamic Light Scattering (DLS)

Dynamic light scattering (DLS) was used to determine the apparent hydrodynamic diameter (D_h_) of the micelles. DLS measurements were carried out at 90° scattering angle on solutions using Autosizer 4800 (Malvern Instruments, Worcestershire, UK) equipped with 192 channel digital correlator (7132) and coherent (Innova, Santa Clara, CA, USA) Ar-ion laser at a wavelength of 514.5 nm. The average diffusion coefficients and hence the hydrodynamic size was obtained by the method of cumulants.

#### 2.1.7. Small Angle Neutron Scattering (SANS)

The SANS experiments were performed using a SANS diffractometer at the Dhruva reactor, Bhabha atomic research centre, Trombay. For SANS, copolymer solutions in D_2_O at different concentrations and temperatures were measured. The solutions were held in a quartz cell of 5 mm thickness with tight-fitting Teflon stoppers. The data were recorded in the Q range of 0.017–0.35 Å. All the measured SANS distributions were corrected for the background and solvent contributions. The data were normalized to the cross-sectional unit using standard procedures [50].

The copolymer micelles consist of a hydrophobic core of PPO surrounded by a hydrated shell of PEO. There is very good contrast between the hydrophobic core and the solvent. However, because of a large amount of D_2_O (water of hydration) being present in the outer PEO corona, the scattering contrast between the hydrated corona and the solvent is expected to be poor. In view of this, we assume that the form factor F(Q) depends only on the hydrophobic core radius. The structure factor S(Q) of the spherical micelles in Equation (1) is calculated using the Percus–Yevick approximation for the case of hard sphere potential in the Ornstein–Zernike equation [51]:

(1) $$\frac{d\Sigma }{d\Omega }\left(Q\right)=nV^{2}{\left(\rho_{P}-\rho_{S}\right)}^{2}P\left(Q\right)S\left(Q\right)+B.$$

The mean core radius (*R*_c_), hard sphere radius (*R*_hs_) and volume fraction (Φ) of the micelles have been determined as the fitting parameters from the analysis. The aggregation number is calculated by the relation *N* = 4πa^3^/3v, where v is the volume of the surfactant monomer.

#### 2.1.8. Solubilization

Drug/dye solubilization measurements were carried out on Shimadzu (UV-2450) UV-Visible double beam spectrophotometers (Tokyo, Japan) with a matched pair of stoppered fused silica cells of 1 cm optical path length. Saturated drug/dye loaded solutions were prepared in glass vessels by mixing excess powdered drug/dye with copolymer solution and stirring at constant temperature at 200 rpm for 2 days. The solutions were filtered (Millipore, 0.45 μm) to remove insolubilized drug/dye. Blank experiments, without copolymer, were done to determine the solubility of the drug/dye in water. The amount of drug/dye solubilized was determined by measuring absorbance at 255/262/470 nm. Calibration with dilute solutions of the drug/dye dissolved in methanol gave satisfactory Beer–Lambert plots. In a solubilization experiment, the filtered solution was diluted thirty times with methanol, the amount of water after dilution being low enough to allow direct use of the calibration plot.

#### 2.1.9. In Vitro Release Studies

The in vitro release of the poorly water soluble anticancer drugs, quercetin and curcumin, from the micelles was investigated using a pre swelled dialysis bag (*M*_w_ cut-off 12,000–14,000 Da). In brief, 10 mL of the drug formulations containing approximately 5 mgs of drugs was transferred into respective dialysis bags and immersed into 100 mL of phosphate buffer saline (PBS), which was placed in a shaking water bath at 37 °C. In addition, 3 mL sample aliquots were taken from the release medium at scheduled time intervals and the same volume of fresh buffer was refilled to maintain the volume. The concentration of drugs released into PBS was quantified based on their absorbance at 470 nm (*λ*_max_ for orange OT), 255 nm (*λ*_max_ for quercetin) and 262 nm (*λ*_max_ for curcumin), respectively using a Shimadzu 160 spectrophotometer on a UV-Vis curve, to further conclude the rate of drug release.

#### 2.1.10. In Vitro Cytotoxicity Assays

Cytotoxicity of free quercetin and curcumin and their loaded micelles of T904 were assessed on the viability of CHO-K1 cells by the 3-(4,5-Dimethylthiazol-2-yl)-2,5-Diphenyltetrazolium Bromide (MTT) assay. These CHO-K1 cells in a logarithmic phase (104 cells/well) were seeded in 96-well plates along with variable concentration of free quercetin and curcumin (0, 12.5, 25, 50 and 100 μg/mL). The cells were also treated with their equivalent doses loaded in T904 micelles. Incubate these cells for 24 h in carbon dioxide incubator (5% CO_2_; 37 °C). Afterwards, add the MTT solution (10 μL; 2.5 mg/mL) into a 96-well plate after centrifuging (2000 rpm; 5 min) and dissolved in an equal volume of media. Again, incubate the plate for another 4 h. Finally, the intracellular formazan crystals were settled at the bottom and the supernatant was discarded (again after centrifuging as mentioned above). These crystals were dissolved in dimethyl sulphoxide and its relative growth inhibition was compared with control cells and its optical density was measured at 570 nm. All experiments were set up in triplicates and repeated thrice for statistical analysis. Results were expressed as mean ± S.E.

The percentage growth inhibition was calculated using the following formula:

$$\%\mathrm{Growth}\;\mathrm{Inhibition}=\frac{100-\left(\mathrm{Mean}\;\mathrm{absorbance}\;\mathrm{of}\;\mathrm{individual}\;\mathrm{test}\right)}{\left(\mathrm{Mean}\;\mathrm{absorbance}\;\mathrm{of}\;\mathrm{Control}\right)}\times 100.$$

The half maximal inhibitory concentration (IC_50_) was estimated using the concentration-response and was expressed in the unit μg/mL. The concentration of the test drug required for the inhibition of cell growth by 50% (CTC50) was generated by the dose-response curves for each cell line.

Statistical Analysis: Data analysis was carried out by two-way ANOVA (SPSS 10.0, SPSS Inc., Chicago, IL, USA). A *p*-value ≤ 0.05 was contemplated as statistically important.

## 3. Results

### 3.1. Characterization of Tetronics^®^ Micelles and the Effect of Salt

#### 3.1.1. Cloud Point

**Figure 2:**
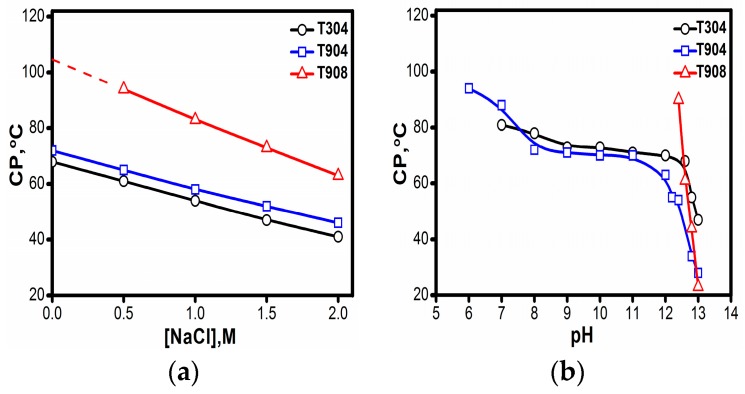
CP of 5% Tetronics^®^ (**a**) as a function of salt concentration and (**b**) pH.

The cloud points (CPs) of all the three copolymers were measured as a function of salt concentration and pH and are presented in Figure 2. It has been established from the previous studies in literature that copolymers fabricated with longer PEO blocks usually exhibit more hydrophilic character and undergo micellization at elevated temperatures. The occurrence of longer PEO blocks improves the solubility of the copolymer chains in water. Hence, these exhibit higher phase separation temperatures. Conversely, those with shorter PEO blocks (>30%) usually display poor solubility and undergo micellization at comparatively lower temperatures and experiences phase separation at relatively lower temperatures [16,44]. In the present case, as displayed in Table 1, T908 with 80% PEO in its constitution remains highly hydrophilic and usually displays CP at temperatures >100 °C in water and, as anticipated, T904 with moderate hydrophilicity with 40% EO in its constitution undergoes phase separation at temperature ~73 °C. Surprisingly, in the case of T304, despite having 40% EO in its constitution like T904, due to low molecular weight of the constituting PPO block, does not form micelles but remains as unimers even at higher temperatures (discussed in later sections) and undergoes phase separation at a temperature very close to that of T904.

For all the copolymers, as shown in Figure 2a, there was almost a linear decrease in CP with an increasing concentration of salt. The presence of salt induces hydrophobicity in the copolymer and makes it prone to form micelles, or micellar growth accounts for the copolymers existing as micelles due to the well-known salting out action of NaCl. A similar CP depressant role of salt has been observed earlier for Pluronics^®^ and other water soluble uncharged polymers that show lower critical solution behavior [52,53,54,55]. Bahadur and coworkers [46,48,49,56] have also reported a similar effect of salt on Tetronics^®^ micelles.

The effect of pH was also examined for these copolymers as shown in Figure 2b. The pH values for the solution were adjusted in the range of 6–13 using HCl/NaOH solutions. For T304 and T904, there is only a slight decrease in CP upon changing pH in the range 6-10; however, a steep fall is seen above pH 10. The CPs were higher in acidic pH because acidic pH makes the amino groups protonated and thus induces a more hydrophilic character and increases CP. For T908, CPs were measured only in the alkaline pH (since its CP in the absence of additives remains >100 °C) where low values of CP were observed. The decrease in CP at higher pH is due to the fact that ethylenediamine group gets completely neutralized at this pH and thus induces more hydrophobicity in the copolymer units promoting micelle formation, which consequently lowers the CP values.

#### 3.1.2. Surface Tension

In order to study the surface activity of these copolymers at the water/air interface and to determine the CMC values, surface tension measurements were carried out as a function of the copolymer concentration in aqueous medium and also in salt solutions in the case of T908.

**Figure 3:**
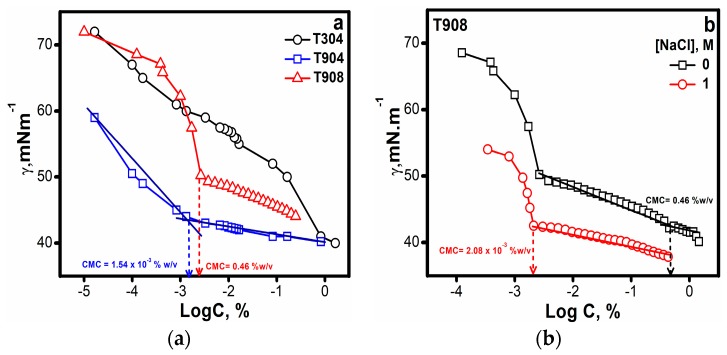
Surface tension plots (**a**) for the aqueous solutions of different Tetronics^®^ and (**b**) T908 solutions in the presence and absence of 1 M salt at 25 °C.

Surface tension → log copolymer concentration plots for the three Tetronics^®^ are shown in Figure 3a. T304 owing to its low molecular weight was least surface active and did not show micelle formation at all the concentrations, as also confirmed through SANS, though a progressive decrease in surface tension (upto 10% *w*/*v*) is observed. On the contrary, Gonzalez-Lopez et al. [20] determined the CMC of T304 in acidic media and observed micelle formation at higher concentrations. T908 with more hydrophilic character was less surface active and showed two break points commonly observed for copolymers with more hydrophilic character. Although improved surface activity and a significant decrease in CMC can be made evident in the presence of salt (Figure 3b). The improved hydrophobicity in the presence of salt is further demonstrated by a single break point (Figure 3b) analogous to moderately hydrophilic copolymers. Conversely, T904 with moderately hydrophilic character displayed highest surface activity among the three copolymers and showed a behavior similar to surfactants. Thus, this leads us to a conclusion that, for all of the copolymers, surface activity increased with the increase in molecular weight, decrease in %EO and in the presence of salt.

#### 3.1.3. Nuclear Magnetic Resonance (NMR)

**Figure 4:**
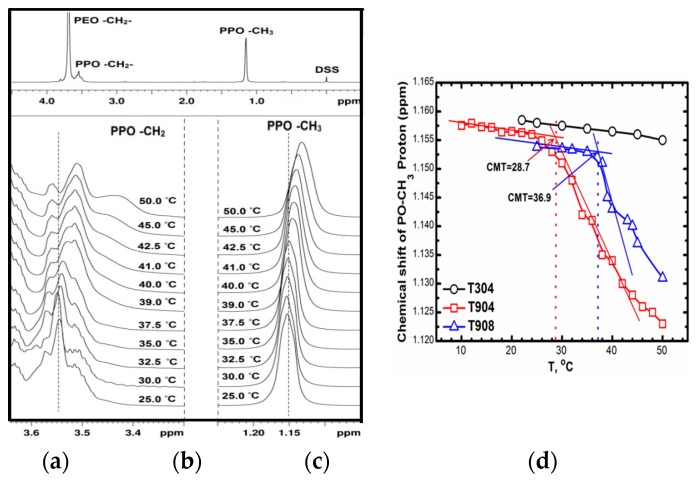
(**a**) ^1^H-NMR spectra of 10% T908 in D_2_O at 25 °C; (**b**) –CH_2_– signals of PPO; (**c**) –CH_3_ signals of PPO; (**d**) chemical shift vs. temperature.

^1^H NMR spectra for the three copolymers were recorded at different temperature intervals and the signals for methylene protons of PEO, methylene and methyl protons of PPO were considered to analyze the spectra (Only spectra for T908 has been included). As evident from Figure 4a, a triplet at 1.16 ppm is assigned to the methyl protons of the PPO, broad peaks around 3.65 to 3.45 ppm correspond to the methylene protons of PPO and intense resonance peak at 3.7 ppm correspond to the methylene protons of PEO. As evident in Figure 4b, an increase in temperature shifts the resonance peaks corresponding to methyl and methylene protons of PPO towards low frequencies with a simultaneous line-width broadening indicating reduced mobility of PPO segments since it forms a hydrophobic core of the micelles.

The chemical shift observed for the PPO –CH_3_ peaks were then plotted against temperature as shown in Figure 4c to determine the critical micelle concentration (CMT) of block copolymers. The CMTs were taken as the inflexion point in the plot. T304 owing to its low molecular weight did not form micelle under the conditions studied despite containing 40% PEO while T904 (with similar %PEO) remains much lower than T908 at the same concentration due to its moderately hydrophobic character. It is interesting to note that T904 and T908 have similar molecular weight of PPO but vary in %PEO, which sufficiently alters their hydrophobicity and micellar and micellization characteristics. A similar trend is also observed for the hydrophilic linear block copolymers, Pluronics^®^ [57,58].

#### 3.1.4. HSDSC

In this study, HSDSC experiments were performed to determine the effect of salt on the CMT of T908. Owing to its hydrophilic character, T908 forms micelles at elevated temperatures as observed in the previous section. Since no micellization was observed in the previous sections for T304, CMT of T304 was not determined. Likewise, measurements for T904, which usually form micelles at lower temperatures, were not carried out since the addition of salt will further decrease the CMT and the practical limitations of the instrument may not allow us to go to such lower temperatures. Hence, the measurements were limited only to T908 to understand the influence of salt on copolymer micelles.

Typical HSDSC thermograms (not shown) with the endothermic peak were obtained signifying the endothermic phase transition from a fully solvated solution of unimers to a solution consisting of solvated micelles with a poly(propylene oxide) microphase inner core. Generally, CMT of the copolymer can be defined by three different methods viz. *T*_onset_, *T*_inf_ and *T*_m_. All three of the methods were explained in detail in our previous reports [44,47]. In the present study, *T*_m_ is chosen as the CMT of the block copolymer.

As evident in data presented in Table 2, the CMT of aqueous solutions of 5% T908 decreases in the presence of salt. This is attributed to the fact that addition of salt leads to the dehydration of the EO and PO blocks, which favors micellization and thus reduces CMT significantly. Alexandridis and Holzwarth [52] using DSC scrutinized the effect of different salts on the CMT of Pluronics^®^ solutions and observed similar results. Similarly, Bahadur et al. [56] using fluorescence witnessed a similar decrease in CMT of T1307 solutions in the presence of salt.

#### 3.1.5. Viscosity

**Figure 5:**
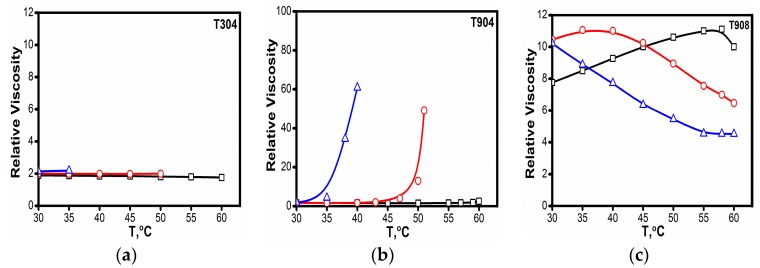
Effect of temperature on different Tetronic^®^ solutions (10% *w*/*v*) in the presence of salt (☐) 0 M, (Ο) 1 M and (△) 2 M NaCl. (**a**) relative viscosity of 10% T304 aqueous and salt solutions versus temperature (**b**) relative viscosity of 10% T904 aqueous and salt solutions versus temperature (**c**) relative viscosity of 10% T908 aqueous and salt solutions versus temperature.

Viscosities of 10% *w*/*v* solutions of Tetronics^®^ in H_2_O, 1 and 2 M NaCl were measured at different temperatures and the relative viscosities were plotted against temperature as shown in Figure 5.

The viscosity behavior of copolymers was different. For copolymer T304, due to its low molecular weight, there was no effect on viscosity and it remained almost constant at different temperatures. The viscosity did not change even in the presence of salt up to its CP. This is because T304 remains molecularly dissolved and does not show any micelles even at elevated temperatures or in the presence of salt. Conversely, T904 with moderately hydrophilic character occurs as micelles at ambient temperature. Any increase in temperature promotes micellar growth due to increased dehydration of EO and PO chains. Hence, more and more unimers participate in micelle formation, eventually leading to micellar growth. Quite interestingly, T908, due to its characteristic hydrophilic character, forms micelles only at high temperature and in the presence of salt. Accordingly, for various concentrations of salts, different morphologies are assumed by the copolymer molecules. In line with this, the contribution from the unimers and micelles shows different viscosity behavior. As evident in the figure, with the increase in temperature, the relative viscosity increases initially but decreases at higher temperatures. This is attributed to the fact that increase in temperature leads to the formation of spherical micelles from the existing unimolecular form of copolymer, which, as a result, enhances the viscosity significantly until a maxima is reached at ~55 °C. As the temperature is raised further, the dehydration of EO blocks is triggered and more compact micelles are formed, which eventually decreases the viscosity of the solution. The presence of salt promotes micellization as also reported in the earlier sections. Hence, the maxima shift to lower temperatures in the presence of 1 and 2 M NaCl. A similar trend has been reported in literature for hydrophilic copolymers earlier. Thus, the results are in line with literature.

#### 3.1.6. DLS

**Figure 6:**
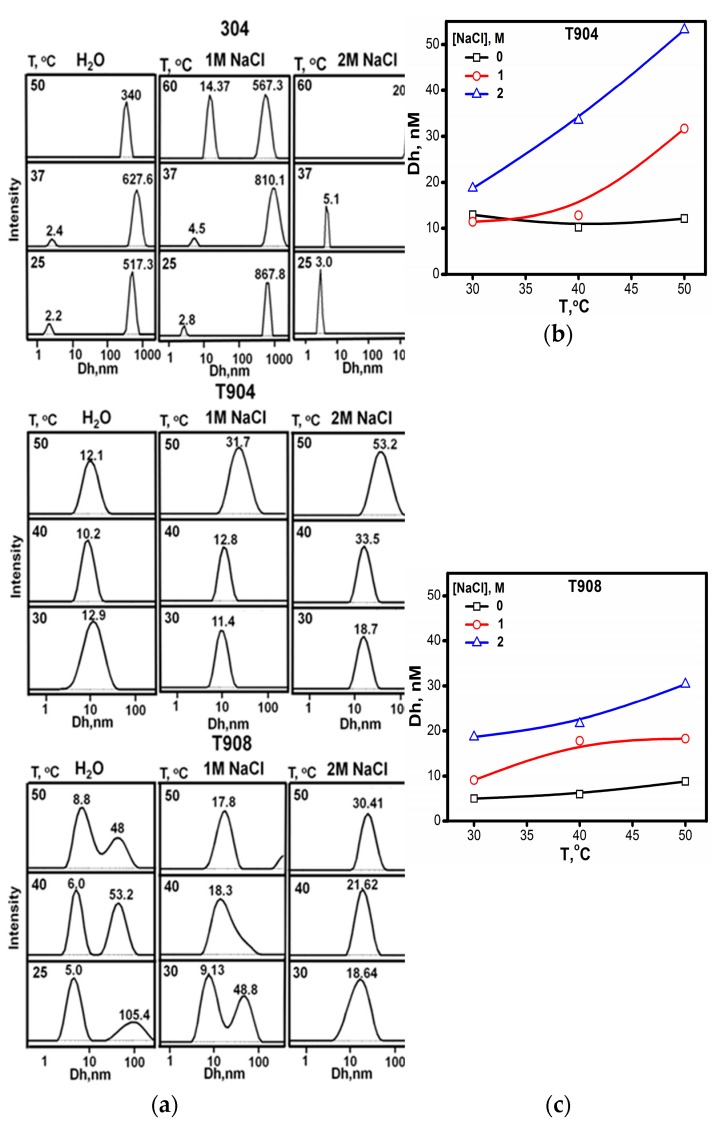
Apparent hydrodynamic diameter of different Tetronics^®^ in aqueous and salt solutions. (**a**) DLS stacks of T304, T904 and T908 in aqueous and salt solutions (**b**) apparent hydrodynamic diameters of aqueous and salt solutions of T904 as a function of temperature (**c**) apparent hydrodynamic diameters of aqueous and salt solutions of T908 as a function of temperature.

The apparent hydrodynamic diameter of micelles at different temperatures for 10% *w*/*v* Tetronic^®^ aqueous and salt solutions was determined using DLS as shown in Figure 6. This figure also displays the effect of temperature (30–50 °C) on the above said copolymer solutions in the presence and absence of salt.

T304 unimer peaks of the size 2–5 nm along with large particles of a few hundred nm are seen. This simply reflects the presence of molecularly dissolved T304 at all temperature and salt concentrations. In the case of T908, the presence of unimers (5–8 nm) along with another peak originating from the presence of micelles and large particles can be clearly seen. With the increase in temperature or in the presence of salt micellar peaks gets sharper while the contribution from large clusters and unimers decreases. Micelles of size ~15–20 nm are the predominant species at higher temperatures and in salt solutions. Single peak arising from the micelles (of ~12–20 nm size) can be seen for moderately hydrophilic T904 at relatively lower temperatures and in the absence of salt. However, micellar growth is observed at elevated temperatures and in the presence of salt. The increased micellar dimensions consequently slow their diffusion in solution, which eventually leads to enhanced solution viscosity.

#### 3.1.7. SANS

**Figure 7:**
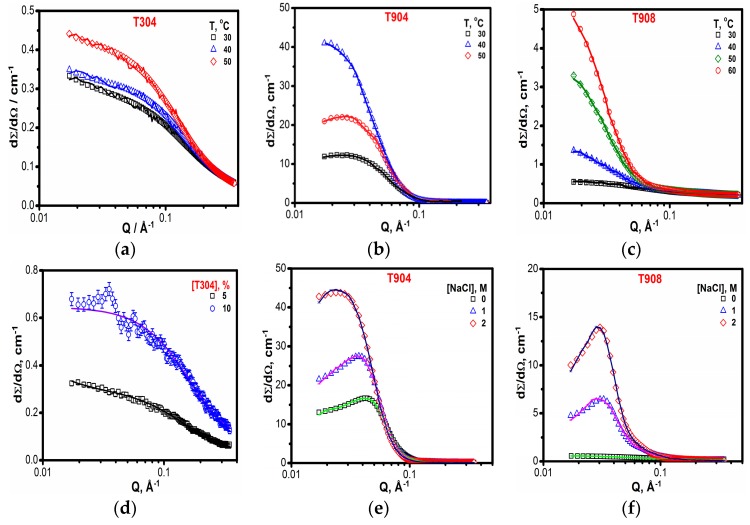
Small angle neutron scattering curves (**a**) for 10% T304 as a function of temperature (**b**) for 5% T904 as a function of temperature (**c**) for 10% T908 as a function of temperature (**d**) for 5% and 10% T304 aqueous solutions (**e**) for 10% T904 aqueous as a function of salt concentration and (**f**) for 10% T908 aqueous solutions as a function of salt concentration.

SANS curves for all three Tetronics^®^ at different temperature are shown in Figure 7. As evident from Figure 7a, the scattering intensity increases with temperature, though overall remains low for T304, indicating an absence of aggregates and the occurrence of only unimers in the solution. This is attributed to the fact that T304, due to its low molecular weight, is incorporated with shorter PPO blocks and hence cannot form micelles. Likewise, T908 with more hydrophilic character occurs as unimers at 30 °C. However, as the temperature is raised, the overall solubility of the copolymer chain decreases, which, in turn, promotes micellization. Thus, as evident in Figure 7c, the scattering intensity of T908 increases significantly as the temperature is raised from 30 to 60 °C. For T904, core-shell micelles were the species present, which show growth at a higher temperature as well as in the presence of salt. It can be observed from Figure 7b that the scattering intensity increases significantly with the increase in temperature, clearly indicating micellar growth.

A similar trend for all three Tetronics^®^ can also be observed from the data presented in Table. In case of T304, it can be understood from the data that neither increase in temperature (Figure 7a) or concentration (Figure 7d) can induce micelle formation and it remains as unimers for all the concentrations and temperatures measured, while, for T904 with moderate hydrophobicity, core-shell micelles with N_agg_ ~ 10 occur in solution at ambient temperature and, with an increase in temperature, R_c_, R_hs_ and N_agg_ increase significantly, clearly indicating micellar growth. In the case of T908, with a more hydrophilic character, only unimers occur at lower temperatures. However, as the temperature is raised, micelles with N_agg_ ~ 7 are formed at 40 °C and an increase in micellar parameters is observed at still higher temperatures, clearly indicating micellar growth at higher temperatures.

The presence of salt has a similar effect and leads to micellar growth at low temperatures. As evident in Figure 7e,f, the increase in concentration of salt improves the scattering intensity indicating growth in micelles. This can be further seen from the calculated parameters shown in Table 3. Thus, SANS results indicate micellar growth in copolymer solutions (except T304) with the increase in temperature/salt concentration in line with the results attained by other techniques.

#### 3.1.8. Micellar Solubilization

**Figure 8:**
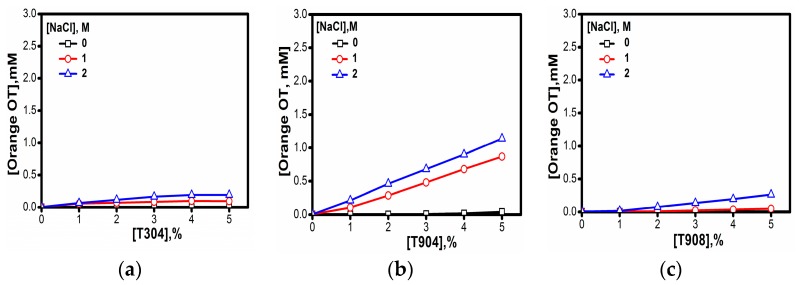
Orange OT Solubility in (**a**) 10% T304 (**b**) 10% T904 and (**c**) 10% T908 aqueous and salt solutions (☐) 0 M, (Ο) 1 M and (△) 2 M NaCl at 30 °C.

To investigate the solubilization behavior of micelles from different Tetronics^®^ in water and salt solutions, one model of hydrophobic dye Orange OT and two drugs—quercetin and curcumin—were taken. As evident in Figure 8, the solubility of the dye increased in the presence of salt. However, for T304, the solubility of the dye was negligible and did not improve in the presence of salt. In contrast, T908 showed poor solubility of dye in the absence of salt, but the solubility marginally increased with the progressive addition of salt. The maximum solubility of the dye was observed for T904 solutions, which further increased many folds in the presence of salt.

**Figure 9:**
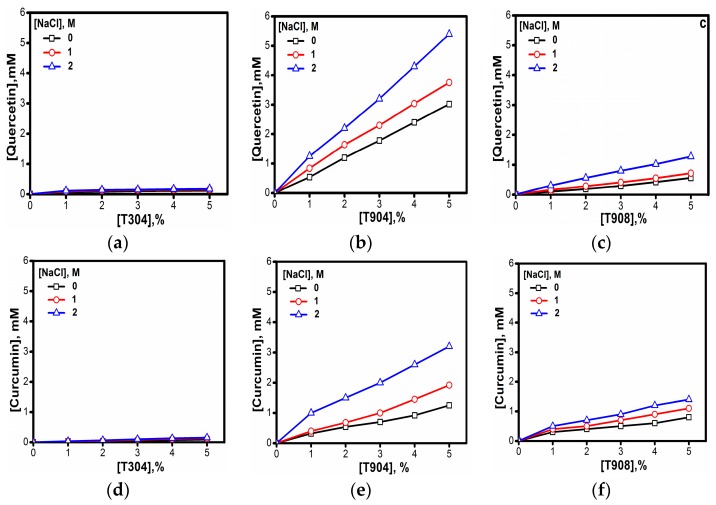
Solubility of quercetin (**a**–**c**) and curcumin (**d**–**f**) in Tetronic^®^ solutions in the presence of (☐) 0 M, (Ο) 1 M and (△) 2 M NaCl at 30 °C.

A similar solubilizing trend of the copolymers was noted for the two poorly water soluble anti-cancer drugs viz. quercetin and curcumin as presented in Figure 9. Pillai et al. [43] compared the solubilizing behavior of the two copolymers T1304 and T1307 in the presence of glycine and observed many fold increase in the solubility of quercetin. The presence of glycine in the above case has an analogous effect to that of salt in the present study. A similar trend in the presence of salt has also been observed by Parekh et al. [59] for the solubility of nimesulide drug by T904 micelles at different copolymer concentrations.

#### 3.1.9. In Vitro Drug Release

In recent years, nanocarrier delivery systems have gained enormous attention for targeting anti-cancer drugs to tumors [60]. Micelles of natural and synthetic biocompatible polymers have been extensively examined as potential carrier materials for drug delivery [61,62,63] by virtue of their dimensions in nano range, competence to shield the encapsulated drug, targeting features by means of the enhanced permeability and retention (EPR) effect [64,65,66], and superior therapeutic capabilities [67]. In addition to their targeting ability, biocompatibility, enhanced circulation time and reduced toxicity are some of the prime factors responsible for the success of these polymers in drug delivery systems. With this approach, in the present work, we have tried to explore the in vitro release behaviors of curcumin and quercetin-loaded T904 micelle formulations under physiological conditions. Since the maximum solubility of the dye and drug in the previous section was observed for the copolymer solutions of T904, the release study was limited only to it.

**Figure 10:**
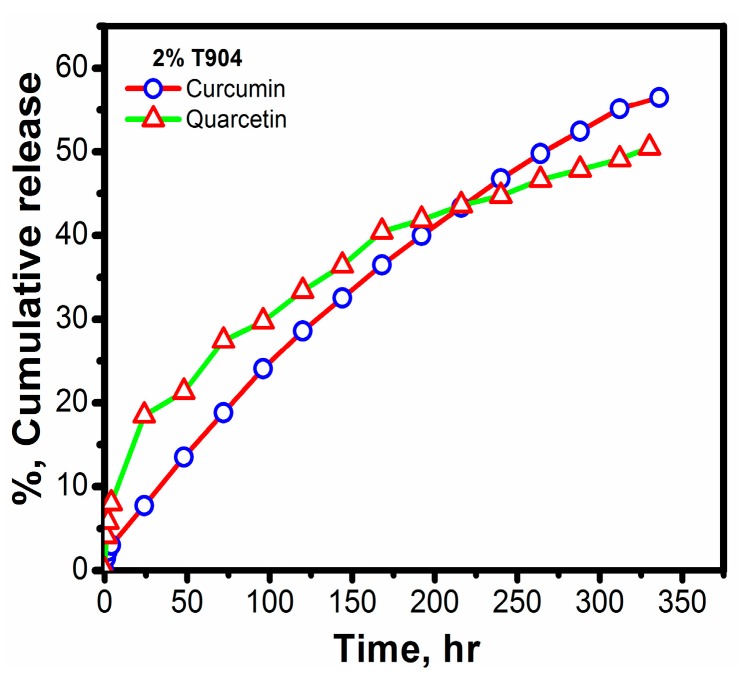
In vitro release profile of (△) quercetin and (Ο) curcumin from T904 micelles at physiological conditions (pH 7.4, 37 °C).

Figure 10 shows the cumulative release profile of two hydrophobic drugs (quercetin and curcumin) versus time where 2% T904 was taken as the release media for understanding its role in in vitro release of both the drugs in physiological conditions. The time intervals were chosen by trial and error method and the study of release was restricted only for the initial 50%. The release remains slow and sustained for both drugs and only about 7% release is observed for curcumin and 18% of release is observed for quercetin during the early 24 h and approximately 50% of curcumin is released in 11 days while for quercetin in about 14 days. This is due to the fact that both the drugs remain localized within the core of the micelle and results in sustained release from this region. 

To understand the drug release kinetics and mechanism, the data was estimated mathematically using zero-order kinetics, first-order kinetics and Higuchi model. The prime condition for choosing the most appropriate model was grounded on best goodness-of-fit (*R*^2^ values). The *k*_0_, *k*_1_, and *k*_H_ values were estimated by fitting the data into corresponding equations and are given in Table 4 along with the regression coefficients (*R*^2^).

From the results shown in Table 4, it is clearly evident that, for T904 micelles, the release of curcumin follows zero order kinetics while that of quercetin follows Higuchi equation. It has been established in literature that Higuchi and zero order kinetics indicate controlled drug diffusion. Hence, we can come to a conclusion that, from T904 micelles, both the drugs were released through diffusion.

#### 3.1.10. In Vitro Cytotoxicity

**Figure 11:**
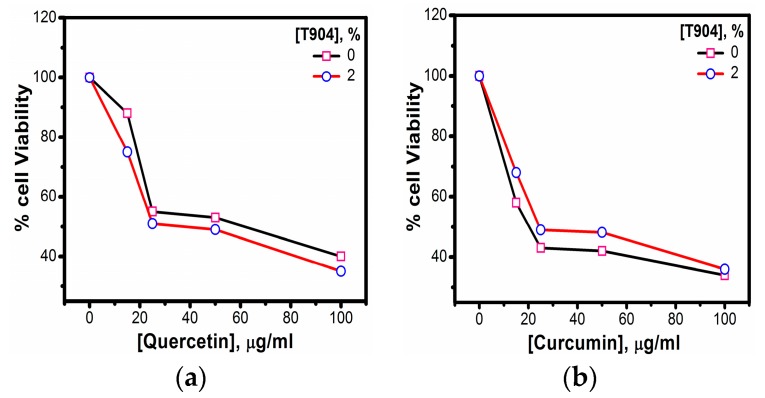
In vitro cell viability of (**a**) quercetin and (**b**) curcumin in the presence and absence of 2% T904.

To investigate the cytotoxicity of curcumin and quercetin from 2% T904 micelles, IC50 was determined on the CHO-K1 cell lines with different concentrations of curcumin and quercetin was evaluated using MTT assay and the data are presented in Table 5. As portrayed in Figure 11, no obvious decline in cell viability was observed after incubation with the blank micelles, indicating that the copolymer was nontoxic for being used as nanocarriers.

To evaluate the feasibility of using this copolymer for cancer therapy, we compared the anticancer effects of free curcumin and quercetin with the drug-loaded T904 micelles. From Figure 11a, it is evident that the quercetin loaded micelles displayed more inhibition of proliferation of CHO-K1 cells than free drugs, while, in the case of curcumin, the IC_50_ of curcumin loaded micelles was comparatively larger than free form. A similar trend has been observed earlier by Pillai et al. [46] for the release of curcumin from the micelles of T1304. According to them, the possible cause for such deviation is due to the difference of drug release rate from micelles inside the cells.

## 4. Conclusions

Aqueous solution behaviour of three star shaped EO–PO block copolymers with different molecular characteristics was examined at different temperature and in the presence of salt using a variety of techniques. The self-assembly was found to be markedly dependent on their molecular characteristics and solution conditions. Data on micellar/phase behavior and interfacial characteristics of the copolymers are reported. More hydrophobic T904 formed micelles at low temperature and showed micellar growth with temperature, T908 with more hydrophilic character does not form micelles at ambient temperature but undergoes micellization at elevated temperatures while T304 with moderate hydrophobicity but low molecular weight remains molecularly dissolved in water in all of the conditions, and does not form micelles even at higher temperatures or in the presence of salt. The presence of salt has an analogous effect to that observed with the increase in temperature. Viscosity/DLS/SANS suggests growth in T904 micelles with temperature. NMR yields CMT, which decreases in the presence of salt. The solubility of dye and drugs improved in the presence of salt and with the increase in copolymer concentration. The in vitro release profiles for both of the drugs showed a slow and sustained release pattern from T904 micelles, and IC50 values for quercetin decreased significantly while increasing for curcumin, probably due to the slow release rate from the core of micelles. Thus, fine-tuning in micellar parameters using the copolymer of desired molecular characteristics and salt at desired temperatures and pH may be useful as nanoreservoirs for delivery systems.

## Figures and Tables

**Table 1:** Structural properties of different Tetronics^®^.

Tetronic^®^	*M*_w_^a^	N_EO_	N_PO_	HLB ^a^	CP ^a^ (°C)	pKa_1_^a^	pKa_2_^a^
T304	1650	3.7	4.3	12-18	72	4.3	8.1
T904	6700	15	17	12-18	78	4.0	7.8
T908	25,000	114	21	>24	>100	5.2	7.9

^a^ Data is taken from BASF website and from references [20,25]. *M*_w_—molecular weight, HLB—hydrophilic-lipophilic balance, CP—cloud point, N_EO_ = y and N_PO_ = x.

**Table 2:** Thermodynamic parameters for micellization of 5% T908 from high sensitivity differential scanning calorimetry thermograms.

[NaCl], M	*T*_onset_ (°C)	*T*_inf_ (°C)	*T*_m_ (°C)	Δ*H* (kJ/mol)	Δ*G* (kJ/mol)	Δ*S* (kJ/mol·K)
**0**	32.00	32.06	37.82	133.38	−26.31	0.51
**1**	19.03	19.73	24.83	178.45	−25.25	0.68
**2**	^a^	^a^	20.26	^a^	−24.80	^a^

^a^ The pre-transitional baseline was so short to calculate the *T*_onset_, *T*_inf_, and Δ*H* properly.

**Table 3:** Micellar parameters of 10% Tetronics^®^ in D_2_O obtained from small angle neutron scattering analysis.

Tetronic^®^	[NaCl], M	Temperature, °C	R_c_, Å	R_hs_, Å	R_g_, Å	N_agg_
**5% T304**	0	30	-	-	10.6	-
**10% T304**	0	30	-	-	11.8	-
**10% T304**	0	35	-	-	11.1	-
**10% T304**	0	50	-	-	13.0	-
**10% T908**	0	30	-	-	25.8	-
**10% T908**	0	40	39.9	-	-	7
**10% T908**	0	50	57.2	-	-	10
**10% T908**	0	60	60.1	-	-	13
5% T904 *	0	30	25.0	52.2	-	10
5% T904 *	0	40	29.9	52.2	-	18
5% T904 *	0	50	33.0	129.0	-	23
**10% T904**	0	30	34.7	51.6	-	24
**10% T904**	1 M	30	39.9	59.6	-	36
**10% T904**	2 M	30	43.61	65.7	-	47
**10% T908**	1 M	30	25.36	81.6	-	8
**10% T908**	2 M	30	44.3	84.9	-	43

* From our previous report [29].

**Table 4:** Different kinetic models describing the release pattern of the anticancer drugs, curcumin and quercetin, from T904 micelles.

Drug	Mathematical Models for Drug Release Kinetics					
	Zero Order		First Order		Higuchi	
	*k*_0_ (M. h^−1^)	*R*^2^	*k*_1_ (h^−1^)	*R*^2^	*k*_H_ (M. h^−1/2^)	*R*^2^
curcumin	0.169	0.99	0.004	0.76	3.321	0.98
quercetin	0.131	0.90	0.002	0.69	2.69	0.99

**Table 5:** The Half Maximal Inhibitory Concentration (IC_50_) of curcumin, quercetin and drug loaded micelles of T904 in the Chinese Hampster Ovarian Cells (CHO-K1 cells).

Solvent	IC_50_, μg/mL	
	Curcumin	Quercetin
H_2_O	18.60	55.2
T904	45.8	48.2
